# Characteristics of Protons Exiting from a Polyethylene Converter Irradiated by Neutrons with Energies between 1 keV and 10 MeV

**DOI:** 10.1371/journal.pone.0157627

**Published:** 2016-06-30

**Authors:** D. Nikezic, Mehrdad Shahmohammadi Beni, D. Krstic, K. N. Yu

**Affiliations:** 1 Department of Physics and Materials Science, City University of Hong Kong, Kowloon, Hong Kong, China; 2 Faculty of Science, University of Kragujevac, Kragujevac, Serbia; ENEA, ITALY

## Abstract

Monte Carlo method has been used to determine the efficiency for proton production and to study the energy and angular distributions of the generated protons. The ENDF library of cross sections is used to simulate the interactions between the neutrons and the atoms in a polyethylene (PE) layer, while the ranges of protons with different energies in PE are determined using the Stopping and Range of Ions in Matter (SRIM) computer code. The efficiency of proton production increases with the PE layer thickness. However the proton escaping from a certain polyethylene volume is highly dependent on the neutron energy and target thickness, except for a very thin PE layer. The energy and angular distributions of protons are also estimated in the present paper, showing that, for the range of energy and thickness considered, the proton flux escaping is dependent on the PE layer thickness, with the presence of an optimal thickness for a fixed primary neutron energy.

## Introduction

For the general public, the majority of the neutron exposure is derived from exposures to cosmic radiation. However, people in certain professions or groups can have higher neutron exposures, including workers at neutron irradiation facilities, nuclear power plants, well loggers, airline crew members, and medical staff and patients involved in clinical radiotherapy. As many of the neutron exposures are long-term, it is pertinent to develop long-term passive methods for monitoring these exposures.

One of the most popular methods for long-term passive measurements of fast neutrons is to employ combinations of converters (to generate protons from neutrons) and proton detectors. As a converter, some hydrogen rich material is used, where elastic scattering of neutrons, i.e., (n,n’p) nuclear reaction is highly probable. The protons obtained from this reaction are then counted with some convenient active or passive device [1,2]. As a converter, polyethylene (PE) with the chemical formula (–CH_2_–CH_2_–)_*n*_ is usually used because of its large contents of hydrogen, low density, low price and convenient mechanical properties. Other converters, like PMMA (C_5_O_2_H_18_) or a combination of (PE + makrofol + nylon) are also in use [3]. In some works, PE is used as a moderator for fast neutrons [4]. Polystyrene has also been used as a converter in combination with PE [5].

The goal of this work is to study in details the protons exiting from a PE layer, in order to assess how the conversion efficiency is dependent upon the layer thickness and the neutron energy. The objective is to investigate in detail the parameters of protons generated within and emerged from polyethylene converters due to neutron irradiation. Such information will be critical in future consideration and development of passive methods for long-term monitoring of neutron exposures.

## Monte Carlo Method Used for Proton Production and Characterization

The Monte Carlo method is used to simulate the neutron interactions within PE, as well as propagation of the generated protons. These will be described in some more details in the text below. Monte Carlo simulations are based on random numbers uniformly distributed in the interval [0,1].

The geometry model is as follows. The neutron beam falls under normal incidence angle on the PE layer. For the sake of simplicity, the neutrons come in the PE layer at the point (0,0,0). The *z*-axis is directed normally on PE along the neutron beam. The protons are “counted” in the simulation on the opposite side of the PE layer, i.e., the surface of the PE layer whose external normal is in the same direction of the incident primary neutron beam. The lateral dimension of the slab has been taken as 1 cm, but an infinite slab can also be defined. No other geometrical details are needed for this simulation.

### Cross sections and ranges

One of the most important parts in this kind of simulation is the cross sections for various processes which can take place when a neutron passes through the PE layer. The cross sections were adopted from the Evaluated Nuclear Data File) (ENDF) library home page: http://www.nndc.bnl.gov/sigma/tree/index.html. The following processes of interaction are taken into account:

- Elastic scattering between neutrons and hydrogen atoms
- Elastic scattering between neutrons and carbon atoms
- Inelastic scattering between neutrons and carbon atoms, including the following reactions: (n,α), (n,3α), (n,γ), carbon excitation on: 4.439 MeV level; 7.6499 MeV level and 9.6399 MeV level.

The ENDF library used in our computer program is ENDF/B-VII.1. The ENDF cross sections employed in our present work have the cut off at 1 keV. The current version of our computer program has the ability to take the incident neutron energy from 100 keV– 10 MeV. Furthermore, the tracked proton energy ranges from 1 keV to a maximum of 10 MeV.

There are other possible nuclear interaction processes with carbon atoms, but their cross sections are zero for the investigated energy range between 1 keV and 10 MeV. For example, the reaction ^12^C(n,d) is also possible but only for energies larger than 14 MeV. Similarly, the ^12^C(n,p) and ^12^C(n,np) reactions are only possible for energies larger than 13.6 and 17.29 MeV, respectively. As such, all these reactions are neglected in the present work.

**Fig 1:**
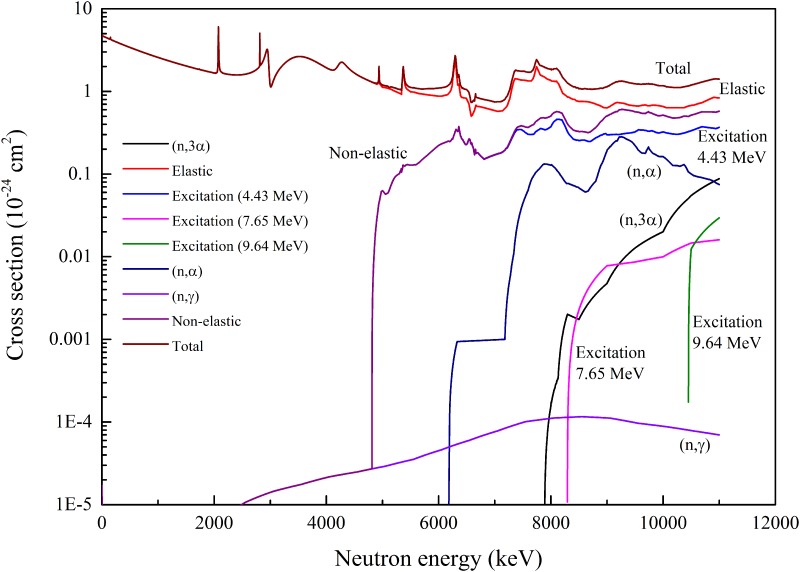
Cross sections for different nuclear reactions between neutrons and ^12^C atoms.

**Fig 2:**
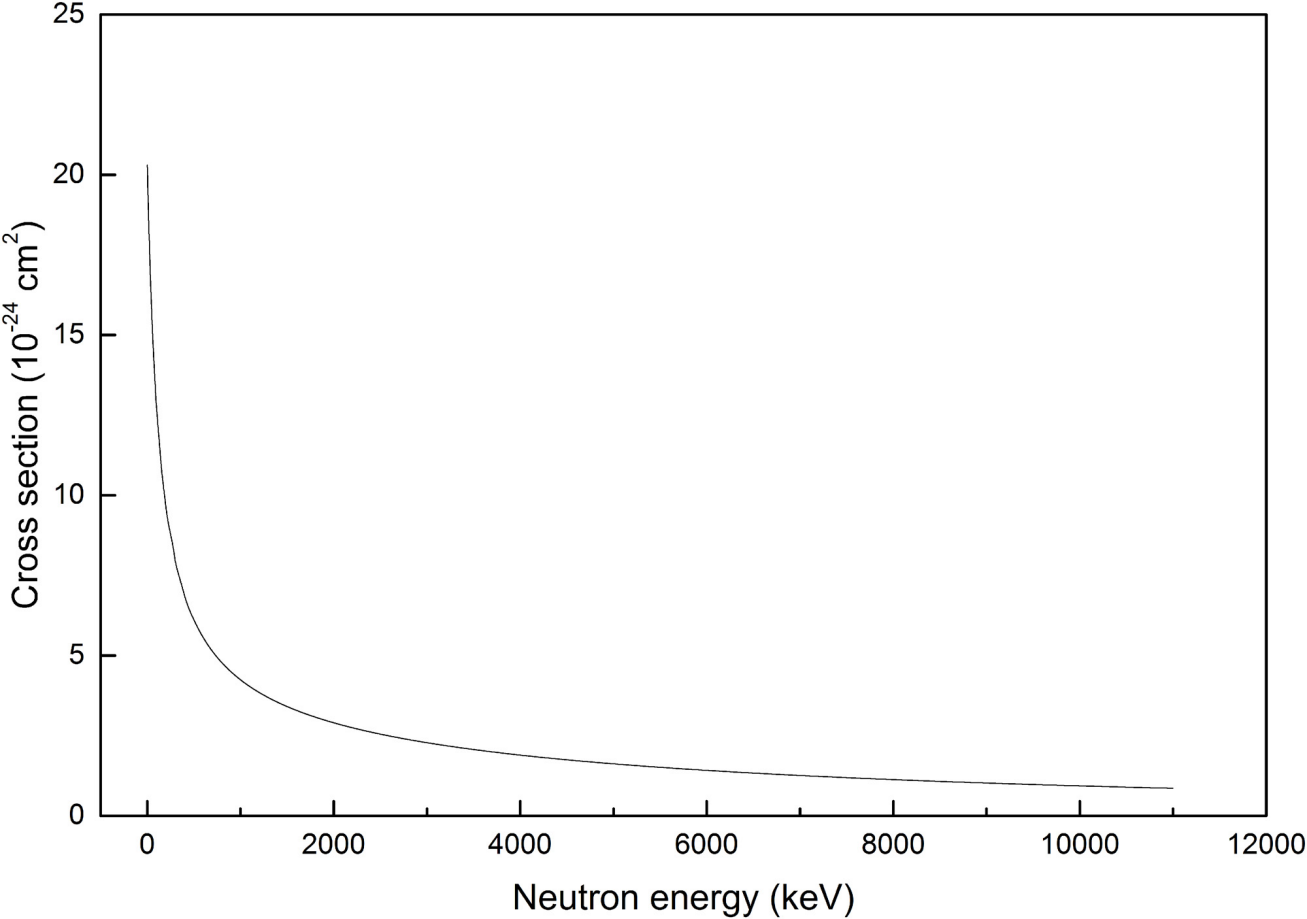
Elastic scattering cross section for hydrogen.

Linear interpolation, with steps of 1 keV, between values given in ENDF has been employed to prepare data sets shown in Figs 1 and 2. In contrast to carbon atoms with which various interactions are possible, only elastic scattering takes place when neutrons interact with hydrogen atoms. The cross sections for this process are shown in Fig 2.

The total macroscopic cross section Σ for interactions between neutrons with polyethylene molecules is obtained through the summation rule as:

$$\Sigma =\frac{\rho N_{Av}}{M}(2\sigma_{C,tot}+4\sigma_{H})$$ (1)

where *ρ* = 0.94 g∙cm^-3^ is the physical density of PE, *N*_*Av*_ is the Avogadro’s number and *M* = 28 is the molar mass of PE, the numbers 2 and 4 are the numbers of carbon and hydrogen atoms in a PE molecule, respectively, and *σ*_*C*,*tot*_ and *σ*_*H*_ are the total cross section of interactions with carbon and hydrogen atoms, respectively.

**Fig 3:**
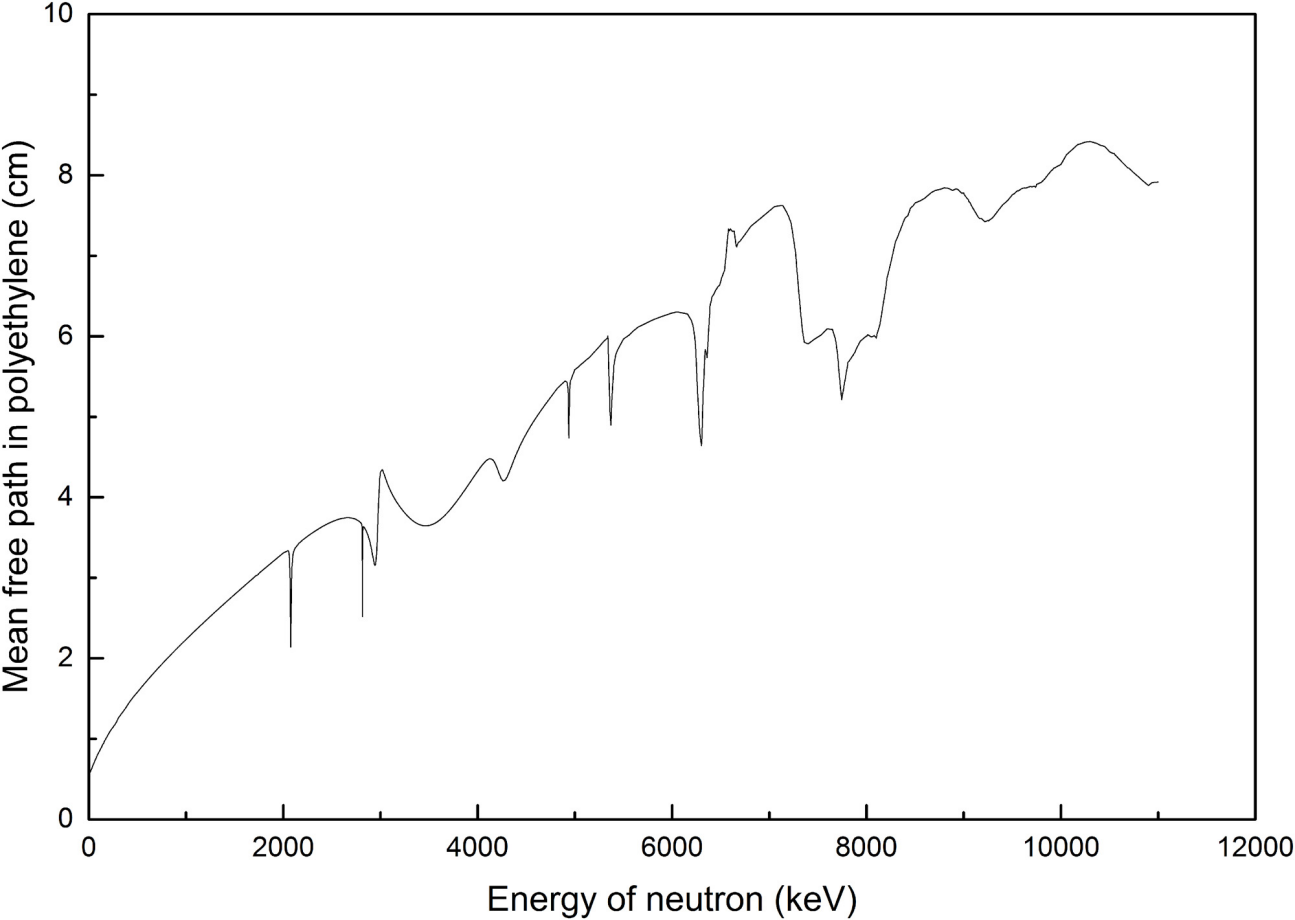
Mean free path for neutrons in polyethylene as a function of the neutron energy.

The mean free path *λ* for neutrons in a material is the reciprocal of the macroscopic cross section, i.e., *λ* = 1/*Σ*, which is shown in Fig 3. A similar graph was previously presented in ref. [6] with less detail.

### Stopping power of protons in polyethylene

**Fig 4:**
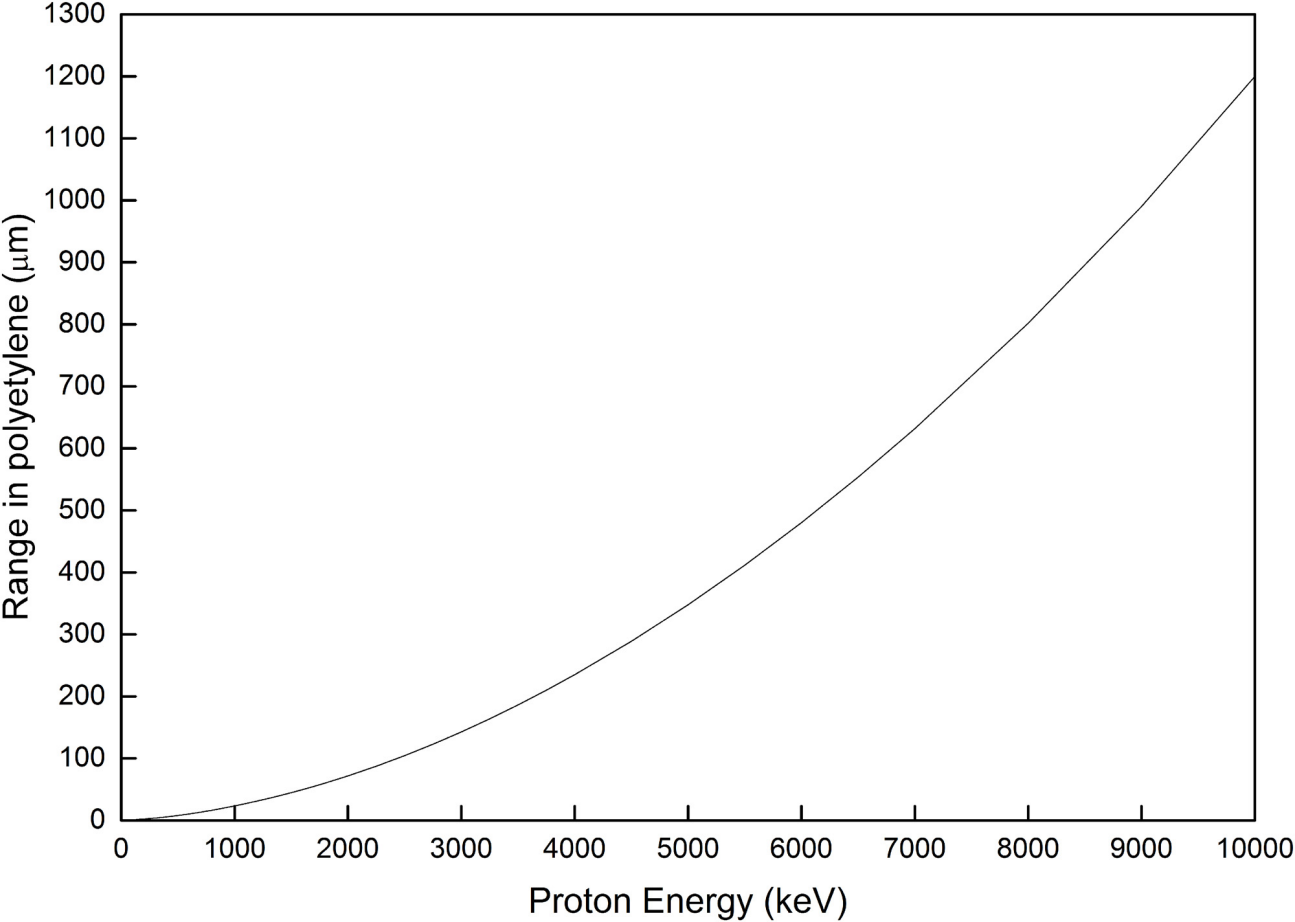
Proton range in PE as a function of the proton energy (obtained using SRIM).

Upon an elastic scattering between a neutron and a hydrogen atom, a recoil proton is generated. Whether the proton will leave the PE layer or not depends on three factors, namely, (1) the position of generation from the surface of the opposite side of the PE layer, (2) direction of recoil, and (3) its range in the PE material. The ranges of protons in PE with different energies are calculated using the Stopping and Range of Ions in Matter (SRIM) computer code and arranged in a convenient format for interpolation calculations [7]. The range of protons in PE is presented in Fig 4.

One can see that the proton ranges are relatively short in PE. The most energetic protons considered in the present study, which have an energy of 10 MeV, have a range of only about 1.2 mm. This means that protons produced by recoils more than 1.2 mm away from the external boundaries have a low probability to escape from the PE layer volume.

### Geometry model

**Fig 5:**
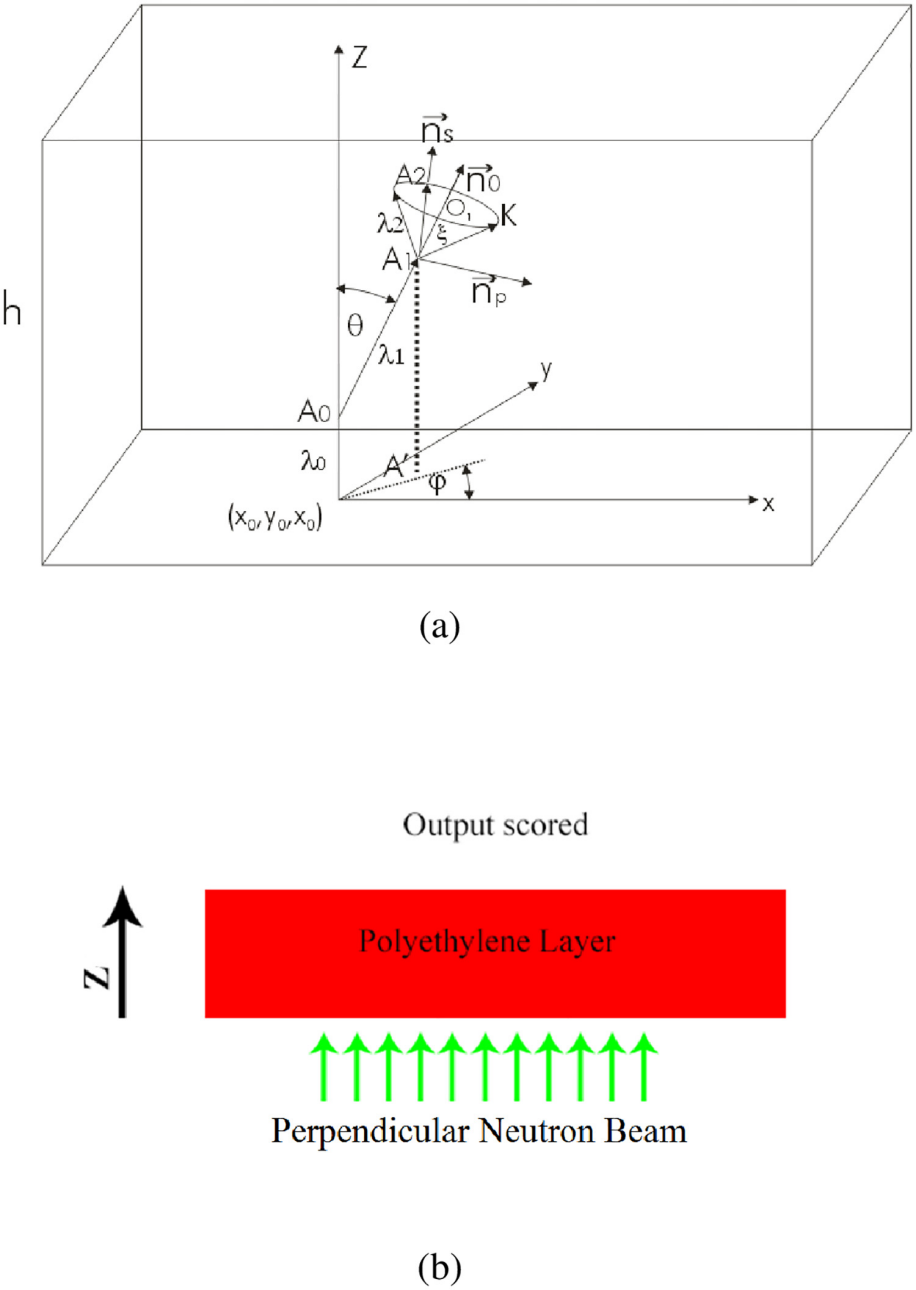
Geometry models and consideration of neutrons beam direction. (a) Geometry of interactions in the PE layer. (b) Schematic diagram showing the neutron beam direction.

We consider the scenario where the neutrons strike the surface of the PE layer with normal incidence. We denote the point of entrance to the PE layer of a neutron with the coordinates (*x*_*0*_ = 0, *y*_*0*_ = 0, *z*_*0*_ = 0). The *z*-axis is defined in the direction which is along the trajectory of the incident neutron, and is thus perpendicular to the surface of the PE layer (see Fig 5b).

The first step in the simulation is to determine free path length *λ*_*0*_ through

$$\lambda_{0}=-\frac{1}{\Sigma }\text{ln}(RN)$$ (2)

where *RN* is a random number (uniformly distributed in the interval [0,1] and obtained by the intrinsic function RANDOM_NUMBER(RN), implemented in Fortran90). The point at which the first interaction occurred is denoted as *A*_*0*_. The interacting atom (C or H) is selected according to the interaction cross section for the given neutron energy. If absorption occurs (for C atoms only), the history has to be terminated and a new neutron is sampled. In the case when a scattering is sampled, the scattering angle *θ* of the neutron is determined in the following way. The energy *E*_*sc*_ of the neutron scattered by a proton is uniformly distributed between 0 and the initial energy *E*_*0*_, so the energy after scattering is sampled as

$$E_{sc}=E_{0}\cdot RN$$ (3)

where *RN* is a new random number. The scattering angle in the laboratory coordinate system of the neutron upon being scattered by a proton is obtained as

$$Cos(\theta )=\sqrt{E_{sc}/E_{0}}$$ (4)

The energy of the recoiled proton is

$$E_{p}=E_{0}-E_{sc}$$ (5)

Moreover, the angle *ψ* of the recoiled proton with respect to the incident trajectory of the neutron (not shown in Fig 5), also in laboratory coordinate system, is

$$Cos\psi =\sqrt{E_{p}/E_{0}}$$ (6)

If the scattering interaction takes place with a carbon atom, the energy *E*_*sc*_ of the scattered neutron is uniformly sampled between *E*_min_ = [(*A*−1)/(*A*+1)]^2^*E*_0_ and *E*_*0*_, where *A* = 12 which is the atomic mass number of carbon. The energy of the recoiled carbon atom is

$$E_{C}=E_{0}-E_{sc}$$ (7)

The scattering angle of the neutron upon being scattered by a carbon atom is given as

$$Cos\theta =\sqrt{\frac{{\left(1+A\right)}^{2}}{4A}\frac{E_{C}}{E_{0}}}\;.$$ (8)

Fig 5 illustrates the situation for which the first interaction takes place at the point *A*_*0*_, and the neutron is scattered through an angle *θ*. In fact, this scattering angle defines a cone with the axis defined by the *z*-axis. Since the problem is symmetrical about the *z*-axis, the angle *φ* is sampled as *φ* = 2*π* · *RN*, where *RN* is another new random number. The angles *φ* and *θ*, as well as the coordinates of the point *A*_*0*_ determined the new traveling direction of the neutron. If a proton is generated by recoil at point *A*_*0*_, its direction will be perpendicular to the new traveling direction of the neutron in the laboratory system.

After each event of scattering, one has to check whether the neutron (and also the proton if generated in that event) exits from the PE layer. If the neutron exits from the PE layer, the history is terminated. If the proton exits from the PE layer, scoring is performed.

A new free path length *λ*_1_ is sampled, and a new interaction takes place at point *A*_1_ (which has a projection *A’* on the *xOy* plane). All the steps described above then has to be repeated, but there is one difference when a neutron is scattered at point *A*_1_ (which has been neglected in the first scattering event) as explained in the following. If the neutron is scattered through an angle *ξ* at point *A*_1_, this angle determines a cone with the axis defined by the traveling trajectory of the neutron before interaction, i.e., connecting the points *A*_0_ and *A*_1_, and represented by the unit vector ${\vec{n}}_{0}$. Sampling of a new direction is not trivial in this case, because the angle *φ* has to be sampled in the plane containing the circle *K* as shown in Fig 5, which has a center at point *O*_1_. The distance *A*_1_*K* is the new free path length defined as *λ*_2_ in Fig 5. As a result, a point *A*_2_ on the circle *K* is sampled, but its coordinates in the *xOy* coordinate system can only be obtained after two rotations and one translation of the coordinate system defined at the point *A*_2_, i.e., a first rotation around the *x*-axis for angle *θ*, followed by a second rotation around the *z*-axis for angle–*φ*, and finally a translation to the point (0,0,0).

If a proton is generated by recoil at point *A*_1_, its direction of motion is found by satisfying the following three conditions:

1. the angle between the scattered neutron direction ${\vec{n}}_{s}$ and the proton direction ${\vec{n}}_{p}$ is π/2, which is expressed as ${\vec{n}}_{p}\cdot {\vec{n}}_{s}=0,$
2. the angle between the initial neutron direction and the proton direction is given by the angle *ψ* (Eq 6), so that ${\vec{n}}_{p}\cdot {\vec{n}}_{0}=Cos\psi$,
3. the conservation of linear momentum, so that the vectors ${\vec{n}}_{p},{\vec{n}}_{0}$ and ${\vec{n}}_{s}$ must be coplanar, i.e., ${\vec{n}}_{p}\cdot ({\vec{n}}_{0}\times {\vec{n}}_{s})=0.$

After the direction of the proton and its initial energy have been determined, with the starting point set at the intersection between the trajectories of the neutron and the hydrogen atom, one should determine the distance *z* between the surface and the starting point (*z* = *h*, where *h* was the thickness of the PE layer). If the distance is larger than the proton range, the proton will be absorbed within the PE layer; otherwise, the proton will exit from the PE layer. In the latter case, the exit energy and the angle (with respect to the normal to the PE layer surface) of the proton are determined and scored for further analyses for the energy and angular distributions, and for other output results.

The interaction with carbon atoms cannot produce recoiled protons with energies in the range considered here. However, interactions between neutrons and carbon nuclei can modify the energy and angular distributions of neutrons within the PE layer. Excitation processes lead to reduction in the neutron energy. As such these processes are taken into account despite their rarity with neutron energies below 10 MeV. After excitation of carbon nuclei, the neutrons are taken to have an isotropic angular distribution but with reduced energies. The (*n*,*γ*) reaction is an absorption process so if it occurs, the neutron history will be terminated. The procedures described above are programed in Fortran90 and executed.

### Benchmarking

We benchmarked our Monte Carlo code with the FLUKA code [8,9] (http://www.fluka.org) by comparing the proton energy distributions for three different PE layer thicknesses of 0.1, 1 and 5 cm for neutron energies of 1 and 10 MeV. The ENDF library used in the FLUKA code is ENDF/B-VI.8.

## Results

### Calculation scheme and outputs

The results presented refer to calculations performed for several thickness of the PE layer, from 0.01 to 5 cm, with energy of the impinging neutrons ranging from 100 keV to 10 MeV.

Several groups of output results are scored and presented here:

- the fraction of neutrons which passes through the PE layer with/without having interactions;
- the energy distribution of protons exiting from the opposite side of the PE layer;
- the angular distribution of protons exiting from the opposite side of the PE layer;
- the efficiency of proton production, i.e., the number of scored protons per one initial neutron.

### Penetration fraction

**Fig 6:**
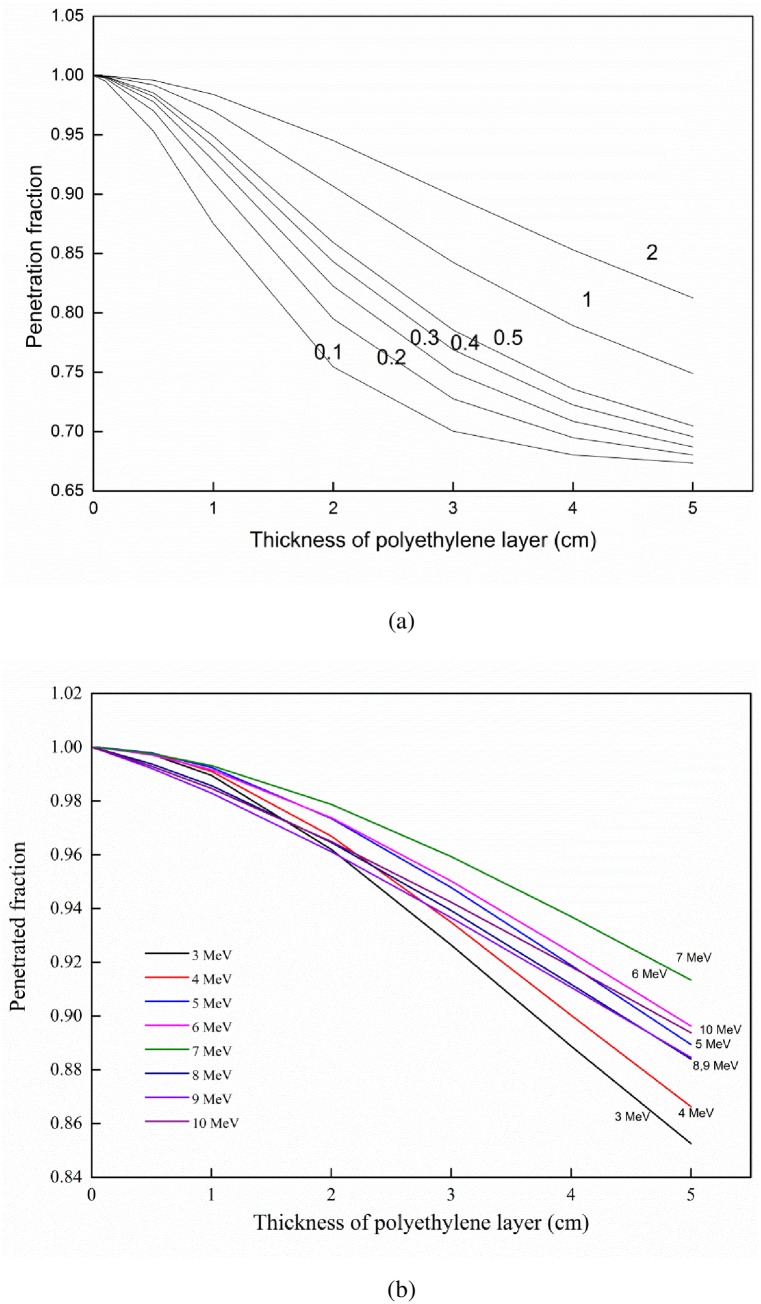
Penetration fraction of neutrons as a function of the PE layer thickness with energy as a parameter. (a) Energy range from 100 keV to 2 MeV. (b) Energy range from 3 to 10 MeV.

Some neutrons pass through the PE layer and exit from the opposite side. The ratio between the number of these neutrons to the original number of neutrons striking the PE layer surface, referred to as the penetration fraction, depends on the thickness of PE layer and the initial energy of the neutrons. These dependences are shown in Fig 6(a) and 6(b).

As expected, the penetration fraction decreases with the thickness of the PE layer. The dependence on energy is regular in the range from 100 keV to 2 MeV, where the penetration fraction increases with energy. However, in the energy range from 3 to 10 MeV, the behavior is more complicated because some of the curves intersect with one another. One should notice that for very thin PE layers (e.g., 0.01 cm), the penetration fraction is almost unity, which means that essentially all neutrons pass through the PE layer. However, this does not mean that they do not experience any interactions. Some neutrons which are scattered can still exit on the PE surface and contribute to the penetration fraction. The penetration fractions of a number of materials used as converters for neutron detection were experimentally measured by El-Khatib et al. [10] who found that the penetration fraction of fast neutrons decreased with the thickness of the PE layer with patterns similar to those shown in Fig 6.

### Efficiency of proton production

The number of protons exiting from the opposite side of the PE layer per one incident neutron is apparently one of the most important parameters which govern the efficiency of a system employing a PE layer as a converter and designed for determining the neutron properties through measuring the properties of the exiting protons.

**Fig 7:**
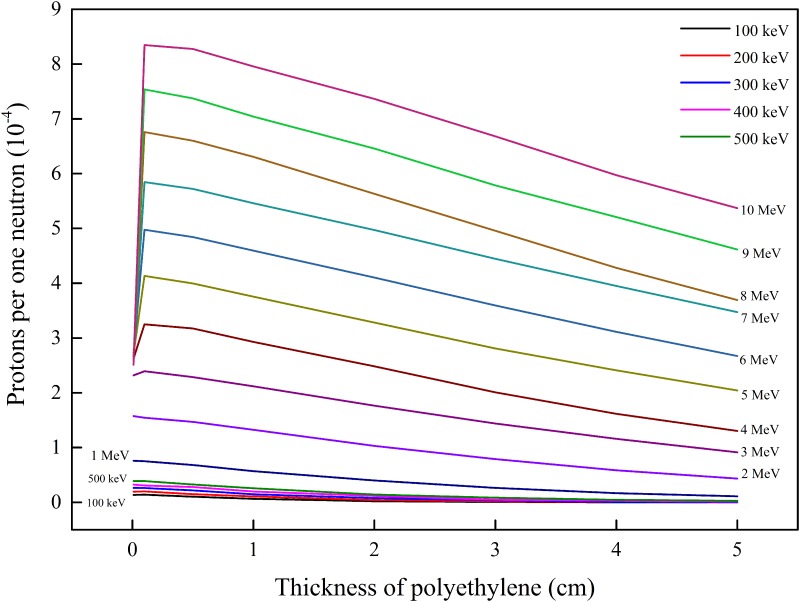
Efficiency of proton production as a function of the PE layer thickness with energy as a parameter (energy range from 2 to 10 MeV).

Fig 7 shows the efficiency of proton production as a function of the PE layer thickness with energy as a parameter (energy range from 2 to 10 MeV). The efficiency of proton production decreases with the thickness of the PE layer, except for the first 0.01 cm. As explained above, the majority of the neutrons effectively just pass through such a thin PE layer without any interaction. Furthermore, it is evident that the efficiency increases with the energy of the neutrons. Neutrons with a smaller energy are less efficient in proton production. These results can be explained by the higher energies of protons produced by the higher-energy neutrons as well as the longer ranges in PE (thus larger chances to exit from the PE layer) for the higher-energy protons.

The abrupt drop in the penetration fraction when the energy of the neutrons increases from 7 to 8 MeV (shown in Fig 6(b)) is explained by the loss of neutron energy through exciting carbon nuclei (7.649 MeV). As a result of this loss of neutron energy, fewer protons can be generated, which is reflected by the closer curves for 7 and 8 MeV in Fig 7 for large PE thickness (when compared with the curves for other energies).

There is an interesting observation in Fig 7 that the maximum proton production per neutron (corresponding to about 1 mm PE thickness) increases almost linearly with the neutron energy when the neutron energy is larger than ~ 5 MeV. This phenomenon is related to the ranges of protons with different energies in PE. Fig 4 shows the relationship between the proton range in PE (calculated using the SRIM computer code) as a function of proton energy. It can be observed that the proton range increases almost linearly with proton energy when the latter is between 5 and 10 MeV. For a PE layer, only protons generated within a distance equal to the range of the protons from the surface of the PE layer can exit from the PE layer. As such, when the neutron energy is between 5 and 10 MeV, the maximum energy of the generated protons, the volume in the PE layer from which generated protons can exit from the PE layer, and thus the output protons will also approximately increase with the neutron energy.

### Energy distributions of produced protons

**Fig 8:**
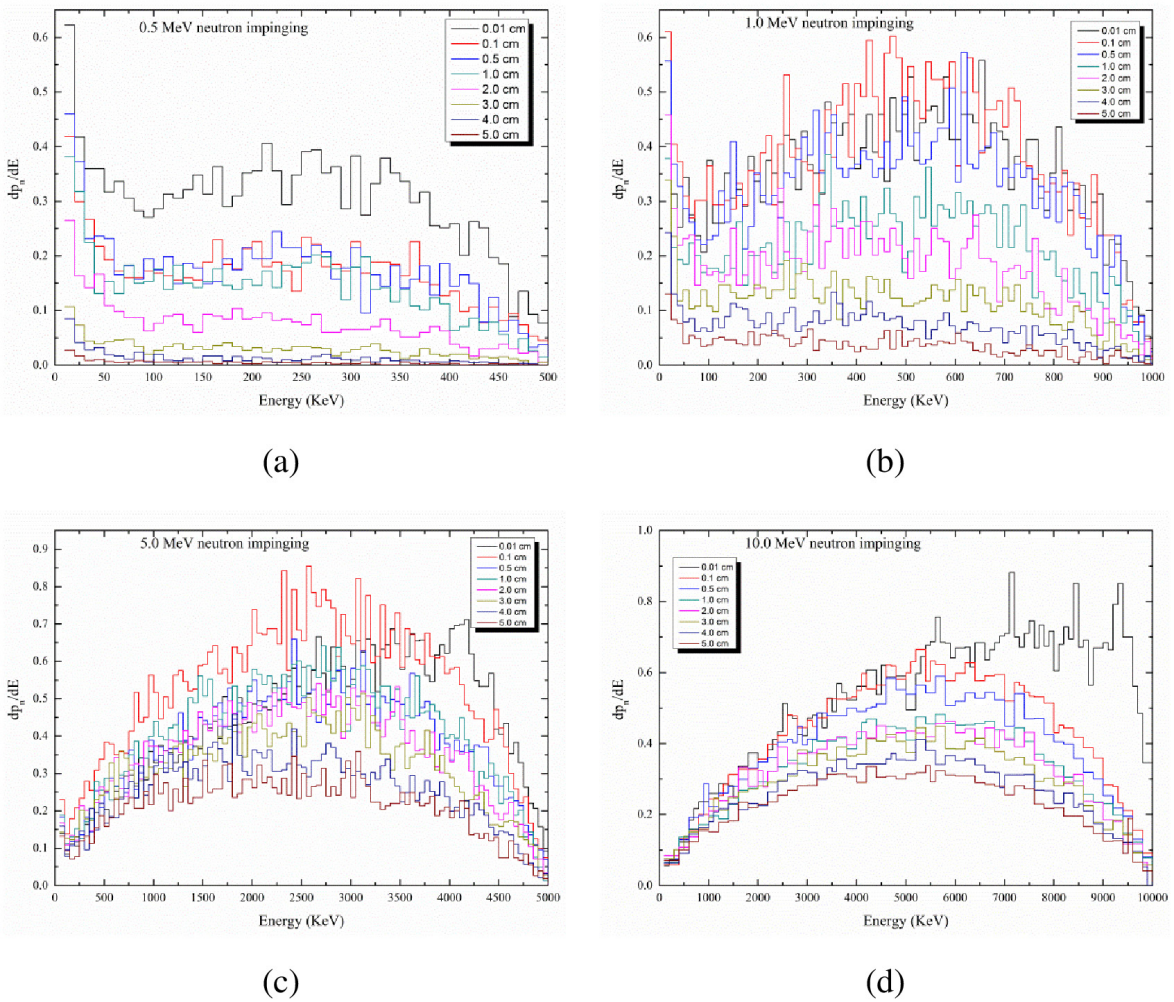
Distribution of protons as a function of the proton energy with the thickness of the PE layer as a parameter (in cm). (a) For incident neutron energy of 0.5 MeV; (b) for incident neutron energy of 1 MeV; (c) for incident neutron energy of 5 MeV; (d) for incident neutron energy of 10 MeV.

Some instruments or techniques for detecting charged particles are sensitive to the energy of the incoming particles so the energy distributions can be important. The energy distributions are studied for the most representative incident neutron energies of 0.5, 1, 5 and 10 MeV in the studied energy range, and the results are shown in Fig 8(a) to 8(d), respectively. These curves are differential energy spectra representing the number of generated protons in intervals of 100 keV, i.e., *dn*_*p*_/*dE*_*p*_.

The proton energy distribution for different polyethylene thicknesses are shown in Fig 8(a) to 8(d) for a variety of neutron energies. It can be observed that *dn*_*p*_/*dE* decreases for thicker PE layers. Moreover, the peak value of *dn*_*p*_/*dE* increases with the neutron energy, while the peaks are more conspicuous for higher neutron energies. For example, *dn*_*p*_/*dE* peaks at about 2500 and 5000 keV for 5 and 10 MeV neutrons, respectively, impinging the PE layer; *dn*_*p*_/*dE* peaks at about 250 and 500 keV for 0.5 and 1 MeV neutrons, but with no noticeable peaks found for thicker PE layers (e.g., 5 cm).

The area under the proton energy distribution curve for a PE layer thickness is mathematically expressed as $\;\int \frac{dp}{dE}dE$, which gives the number of protons escaping per unit neutron for that particular PE layer thickness. The results in Fig 8 show that the number of protons escaping per unit neutron increases with the neutron energy, but decreases with increasing thickness for the PE layer. In addition, the obtained results on the proton energy distribution are in excellent agreement with the results from simulations using the FLUKA code (see the section on “Benchmarking”).

### Angular distributions of produced protons

**Fig 9:**
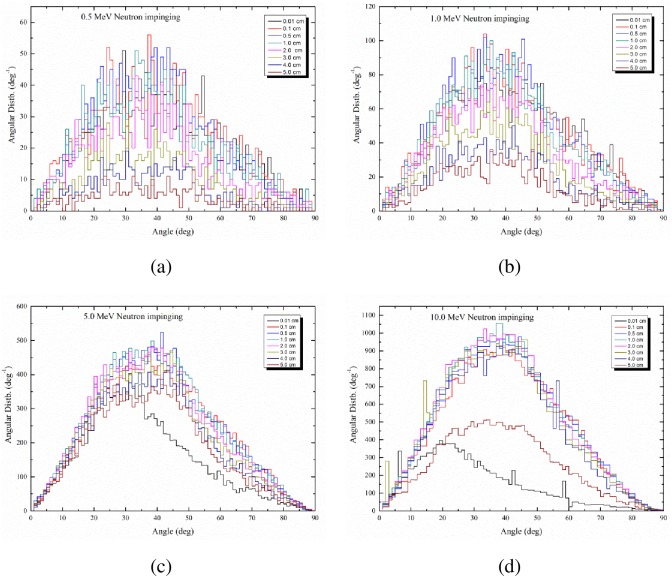
Distribution of protons as a function of the angle with the thickness of the PE layer as a parameter (in cm). (a) For incident neutron energy of 0.5 MeV; (b) for incident neutron energy of 1 MeV; (c) for incident neutron energy of 5 MeV; (d) for incident neutron energy of 10 MeV.

Some instruments or techniques for detecting charged particles are sensitive to the incident angle of the incoming particles. One example is the solid-state nuclear track detectors (SSNTDs) such as the polyallyldiglycol carbonate (marketed as CR-39) and cellulose nitrate (marketed as LR 115) detectors, in which there is a so-called critical angle of detection [11]. Particles which enter the SSNTD with an incident angle smaller than the critical angle will not develop visible nuclear tracks in the detector and thus will not be detected [11]. The angular distributions are studied for the most representative incident neutron energies of 0.5, 1, 5 and 10 MeV in the studied energy range, and the results are shown in Fig 9(a) to 9(d), respectively.

The proton angular distribution for different incident neutron energies and PE layer thicknesses are shown in Fig 9(a) to 9(d). The area under a curve decreases with increasing PE layer thickness. For neutrons perpendicular impinging the PE layer, a peak is in general observable at approximately 20° to 25°, and the peak shifts to angles smaller than 20° for higher neutron energies. Furthermore, the area under a curve increases with the neutron energy. In fact, $\int_{\theta min}^{\theta max}\frac{dp}{d\theta }d\theta$ gives the number of protons escaping from the system, so we can conclude that this number is dependent on the neutron energy and the PE layer thickness. However, for high neutron energies such as 5 and 10 MeV, the number of protons escaping from a PE layer with a thickness of 0.01 cm is much lower when compared to other thicknesses. This shows that for high energy neutrons, there will be much fewer interactions for very thin PE layers.

Mackovicka et al. [12] also examined the angular and energy distributions of protons produced in the PE layer by neutron irradiation, but only studied a PE layer thinner than 0.3 mm and only considered elastic scattering. The possible multiple scattering of neutrons which would be important for thicker converters were not taken into account. Despite these limitations, the authors obtained distributions with similar shape to those obtained in the present work, although these were quantitatively different as expected. The authors also demonstrated the increase in the efficiency of proton production with the neutron energy, but again the numerical values were not commensurate with those obtained in the present work.

### Benchmarking results

**Fig 10:**
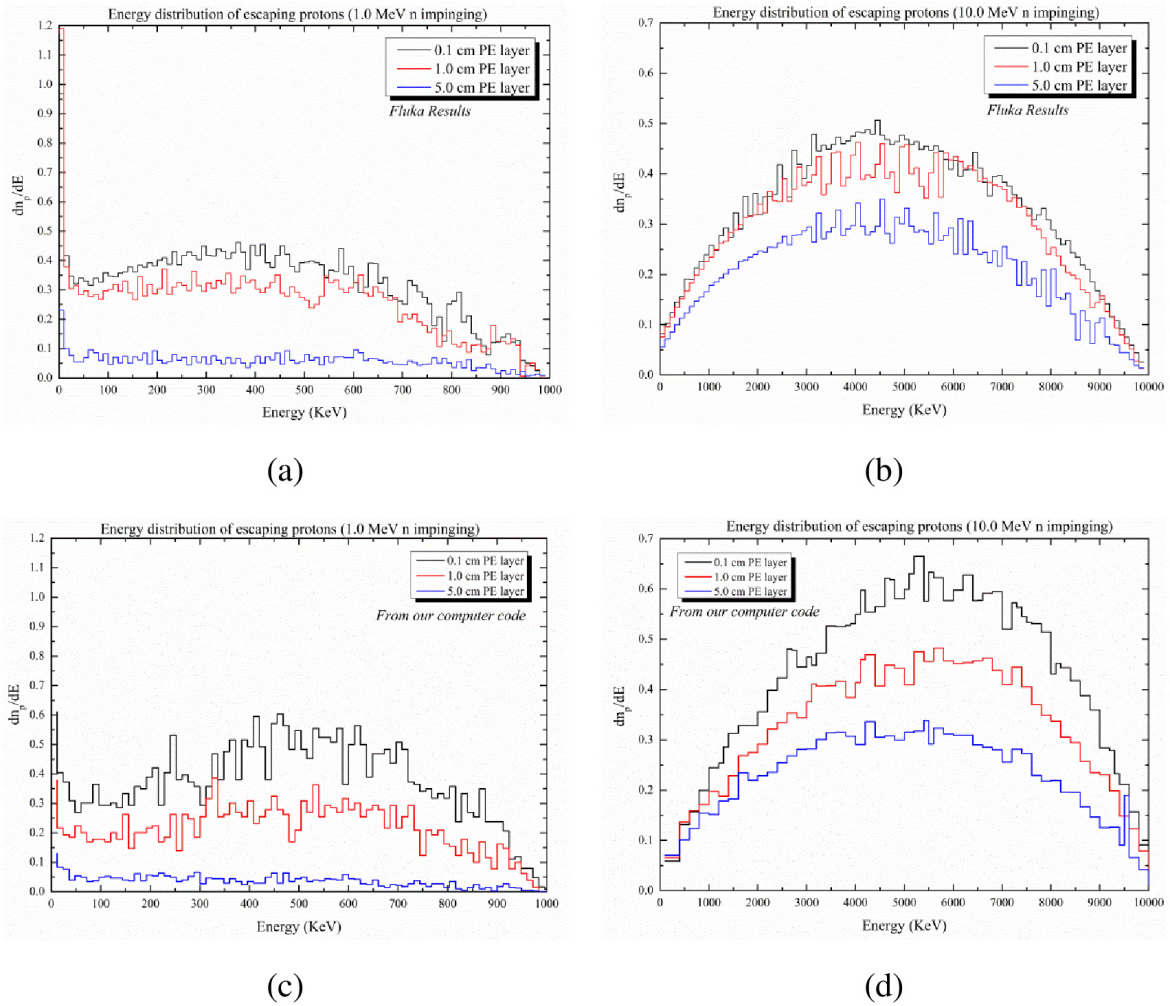
Comparison between the energy distributions of protons exiting from the opposite side of a PE layer obtained using the FLUKA code and our present code. (a) FLUKA results for 1 MeV neutrons impinging PE layer; (b) FLUKA results for 10 MeV neutrons; (c) Results from our computer code for 1 MeV neutrons; (d) Results from our computer code for 10 MeV neutrons.

We have compared the results on the proton energy distribution from using the FLUKA code (shown in Fig 10(a) and 10(b)) and from using our computer code (shown in Fig 10(c) and 10(d)), which show excellent agreement. Here, we employed similar geometries with equivalent dimensions in the FLUKA code and in our computer program, and scored the energy spectra of escaping protons using 10^4^ incident neutrons in both computer codes. The minor differences are mainly due to differences in the employed cross-section data.

## Conclusions

The characteristics of protons produced by neutron irradiation of polyethylene (PE) converters have been studied in detail in the present work. The energy range of neutrons studied is between 1 keV and 10 MeV. Several conclusions can be drawn from the present results:

1. The efficiency of proton production is between 10^−6^ and 10^−3^, increases with the neutron energy, and slightly decreases with the thickness of the PE converter. A very thin PE layer (0.01 cm) is inefficient in proton production because most neutrons pass through it without any interactions.
2. The penetration of neutrons through the PE layer is large, and decreases with the thickness of the PE layer. Furthermore, the penetration of neutrons decreases with the PE layer thickness with some irregularities for neutron energies larger than 7 MeV, which is caused by large reductions in the neutron energies due to excitation of carbon atoms.
3. The energy distribution of protons depends on the PE layer thickness and the initial neutron energy. The peak values for the proton energy distribution increases when the PE layer thickness decreases or when the neutron energy increases.
4. The angular distribution of protons also depends on the PE layer thickness. The area under an angular distribution gives the number of protons escaping from the system. From the obtained results, we conclude that the number of protons escaping increases when the PE layer thickness decreases or when the neutron energy increases.

These results provide valuable information for future consideration and development of passive methods for long-term monitoring of neutron exposures.
